# Arbuscular mycorrhizal fungi alter above- and below-ground chemical defense expression differentially among *Asclepias* species

**DOI:** 10.3389/fpls.2013.00361

**Published:** 2013-09-19

**Authors:** Rachel L. Vannette, Mark D. Hunter, Sergio Rasmann

**Affiliations:** ^1^Biology Department, Stanford UniversityStanford, CA, USA; ^2^Department of Ecology and Evolutionary Biology, University of MichiganAnn Arbor, MI, USA; ^3^Department of Ecology and Evolutionary Biology, University of LausanneLausanne, Switzerland

**Keywords:** plant–herbivore interactions, mycorrhizal fungi, plant defense, above-below-ground interactions, growth-defense tradeoff, root defense, phylogenetic signal

## Abstract

Below-ground (BG) symbionts of plants can have substantial influence on plant growth and nutrition. Recent work demonstrates that mycorrhizal fungi can affect plant resistance to herbivory and the performance of above- (AG) and BG herbivores. Although these examples emerge from diverse systems, it is unclear if plant species that express similar defensive traits respond similarly to fungal colonization, but comparative work may inform this question. To examine the effects of arbuscular mycorrhizal fungi (AMF) on the expression of chemical resistance, we inoculated 8 species of *Asclepias* (milkweed)—which all produce toxic cardenolides—with a community of AMF. We quantified plant biomass, foliar and root cardenolide concentration and composition, and assessed evidence for a growth-defense tradeoff in the presence and absence of AMF. As expected, total foliar and root cardenolide concentration varied among milkweed species. Importantly, the effect of mycorrhizal fungi on total foliar cardenolide concentration also varied among milkweed species, with foliar cardenolides increasing or decreasing, depending on the plant species. We detected a phylogenetic signal to this variation; AMF fungi reduced foliar cardenolide concentrations to a greater extent in the clade including *A. curassavica* than in the clade including *A. syriaca*. Moreover, AMF inoculation shifted the composition of cardenolides in AG and BG plant tissues in a species-specific fashion. Mycorrhizal inoculation changed the relative distribution of cardenolides between root and shoot tissue in a species-specific fashion, but did not affect cardenolide diversity or polarity. Finally, a tradeoff between plant growth and defense in non-mycorrhizal plants was mitigated completely by AMF inoculation. Overall, we conclude that the effects of AMF inoculation on the expression of chemical resistance can vary among congeneric plant species, and ameliorate tradeoffs between growth and defense.

## Introduction

Plants are preyed upon by a diverse community of enemies, including pathogens and herbivores, which damage plant tissue both above- (AG) and below-ground (BG) and can severely reduce plant fitness. In response, plants have evolved a diversity of defenses, including the ability to tolerate or resist such attack (van der Meijden et al., 1988; Karban and Baldwin, 1997). We focus here on plant resistance to herbivory, which includes the expression of toxic secondary compounds, those that reduce nutrient assimilation by herbivores, or even attract predators of such herbivores. Although specialist herbivores exhibit adaptations to ameliorate the effects of toxins produced by their host plants, these same toxins can also negatively affect herbivore growth. For example, plants in the genus *Asclepias* produce potent cardenolides, and although specialist herbivores, including *Danaus plexippus* and *Tetraopes tetraophthalmus*, can tolerate and sequester these compounds (Agrawal et al., 2012), high concentrations or novel combinations of cardenolides can reduce performance on milkweed hosts (Zalucki et al., 2001b; Rasmann and Agrawal, 2011a). As a result, variation in the expression of defense compounds by plants, whether driven by genotype or phenotypic plasticity, can influence the performance of both specialist and generalist herbivores.

Classical theories of plant defense expression AG describe how both the expression and evolution of defense compounds depend on the abiotic environment and levels of herbivory (Bryant et al., 1983; Coley et al., 1985; Herms and Mattson, 1992). However, recent evidence indicates that the presence and abundance of microorganisms may also affect the expression of plant defenses AG and BG (Rieske, 2001; Thamer et al., 2011). Microbial symbionts in roots, for instance, may affect plant resistance systemically through a number of hormonal, nutritive, or signaling pathways (Vannette and Hunter, 2009; Pineda et al., 2010). Although plants in natural settings are nearly always associated with such microorganisms (Smith and Read, 2008; Partida-Martinez and Heil, 2011), the consequences of plant-microbe interactions are rarely incorporated into plant defense theories (but see Bennett et al., 2006; Vannette and Hunter, 2011). As a result, understanding microbial effects on plant resistance and plant–herbivore interactions should have broad implications for our understanding of both the evolution and expression of plant defense.

We focus here on plant associations with Glomeromycetes, also known as arbuscular mycorrhizal fungi (AMF). These ubiquitous symbionts colonize the roots of the majority of land plant species examined for the association (Smith and Read, 2008), often increasing plant phosphorus and nitrogen uptake in exchange for carbon transferred to the fungus. This exchange leads directly to predictions for the expression of nutrient-limited defenses (Vannette and Hunter, 2011) and implies that AMF may modify the shape of the theorized growth-defense tradeoff (De Deyn et al., 2009; Vannette and Hunter, 2011). A growing number of studies have examined the influence of AMF on plant defense expression and herbivory, and a few trends have emerged. For example, inoculation with AMF often increases the performance of specialist chewing insects, and decreases the performance of generalist insects (Gehring and Whitham, 1994; Koricheva et al., 2009). In one of the few studies to link the expression of resistance traits to herbivore performance, suppression of AMF in the roots of *Plantago lanceolata* decreased the concentration of two major iridoid constituents in leaves (Gange and West, 1994). Reductions in chemical defense in *P. lanceolata* were associated with significant increases in consumption by the generalist folivore *Arctia caja*. Despite the potential importance of plant-associated microbes for plant–herbivore interactions, their effects on plant defense are hardly generalizable. For example, AMF species differentially affect expression of iridoid glycosides in *P. lanceolata* (Bennett et al., 2009) and of cardenolides in *Asclepias syriaca* (Vannette and Hunter, 2011). However, when AMF identity is kept constant, does plant resistance among species respond similarly to AMF inoculation? One study reported that inoculation with *Glomus intraradices* increased the growth of the insect herbivore *Spodoptera littoralis* to an equivalent degree among 6 unrelated grassland plants (Kempel et al., 2010), although the mechanism of effect was not investigated. However, a field study demonstrated high levels of variation among prairie plant species in tolerance to herbivory in the presence of AMF (Kula et al., 2005). Similarly, AMF inoculation differentially affected several genotypes of *Datura stamonium* in their level of tolerance to herbivory (Garrido et al., 2010), and full-sibling families of *Plantago lanceolata* also responded differently in the expression of iridoid glycosides with AMF inoculation (De Deyn et al., 2009). However, relatively few studies actually examine the effects of AMF on the expression of resistance among congeners. As a result, predicting the outcome of multitrophic interactions based on plant identity remains a challenge.

In order to incorporate the effects of AMF on plant defense into ecological and evolutionary theory, we should examine whether inoculation with mycorrhizal fungi affects similarly plants that share defense compounds. Previous work using *A. syriaca* demonstrates that an increase in AMF abundance is often associated with increased plant phosphorus status and with expression of phosphorus-limited defenses, including cardenolides and trichomes (Tao and Hunter, 2012; Vannette and Hunter, 2013). Will such patterns hold among other species of *Asclepias*? Plant species vary in the degree to which their phenotypes respond to AMF (Klironomos, 2003; Janos, 2007) and this may include their chemical resistance traits.

In addition, given the importance of root-feeding herbivores in both natural and managed ecosystems (Hunter, 2008), increasing efforts to study root defense have demonstrated that putative plant resistance traits in roots significantly influence BG herbivore performance (Rasmann and Agrawal, 2011a). AMF colonization may also further increase plant protection against BG feeders (Gange and West, 1994; Vannette and Rasmann, 2012). However, the expression of secondary compounds BG could also negatively affect mycorrhizal associations (Strauss and Irwin, 2004). As a result, balancing the potential benefits of root defense and mycorrhizal gains may pose a tradeoff for many plants. Additionally, initial results suggest that mycorrhizal fungi may differentially affect AG and BG defense (De Deyn et al., 2009), but the effects of AMF on the relative expression of resistance compounds between AG and BG tissues is unclear. Such effects could be ecologically relevant, with different consequences for the performance of shoot and root feeders.

To examine the effects of AMF on plant resistance both AG and BG, we assessed how mycorrhizal inoculation affects the expression of cardenolides, a class of steroid glycosides, among eight *Asclepias* species. Here, we used a comparative phylogenetic approach to disentangle factors that generate variation in AMF-mediated multitrophic interactions. Phylogenetic methods provide powerful tools with which to compare species-specific responses within single experiments. In this case, while controlling for relatedness, we can consider whether plant responses to AMF are predictable within a single clade. Specifically, we asked the following questions: first, does inoculation with mycorrhizal fungi differentially influence the expression of defense compounds among congeneric plant species? Second, does association with AMF influence the distribution of defense compounds among plant roots and shoots? Third, are the effects of AMF on plant biomass and defense similar in AG and BG tissues? Finally, do AMF affect the relationship between plant growth and the expression of secondary chemistry?

We used milkweeds, in the genus *Asclepias*, to examine the effects of AMF inoculation on the expression of plant resistance compounds in roots and foliar tissue. Plant species in the genus *Asclepias* are an ideal system with which to address this question, as nearly all species produce cardenolides, toxic molecules which can disrupt the sodium and potassium flux in animal cells when ingested (Malcolm, 1991). These compounds occur in all milkweed tissues, including roots (Rasmann et al., 2009), and have selected for a community of specialist herbivores shared among *Asclepias* species. Despite insect behavioral and physiological adaptations to reduce cardenolide exposure and toxicity (Dussourd and Eisner, 1987; Holzinger and Wink, 1996), empirical evidence suggests that cardenolides continue to be detrimental to both AG (Zalucki et al., 2001a; Agrawal, 2005; de Roode et al., 2011) and BG herbivores (Rasmann et al., 2011). *Asclepias* species are also colonized by mycorrhizal fungi, which can influence growth and the expression of resistance phenotypes, including cardenolide expression (Vannette and Hunter, 2011; Vannette and Rasmann, 2012).

## Materials and methods

### Experimental design

In order to assess the influence of AMF on AG and BG expression of defense among *Asclepias* species, we selected eight species all producing variable levels of cardenolides (Rasmann and Agrawal, 2011b), and grew them in the presence and absence of mycorrhizal fungi. Because of high phylogenetic signal for cardenolide production across the American milkweeds (Agrawal and Fishbein, 2008), the species were also chosen in order to cover most of the phylogenetic variation. Additionally, all eight species are known to be associated with root herbivores, the longhorn beetle larvae in the genus *Tetraopes* (Farrell and Mitter, 1998), live in very distinct habitats (Rasmann and Agrawal, 2011a), and associate with mycorrhizal fungi (Vannette and Rasmann, 2012).

Plants were grown as described previously (Vannette and Rasmann, 2012). Briefly, seeds were cold stratified and germinated in petri dishes. Individual seedlings (*n* = 10−26 replicates per plant species depending on germination success) were transplanted into 10 cm diameter plastic pots in a mixture of low nutrient, autoclaved potting soil (Metro-Mix 360, Metro-Mix Sun Gro Horticulture Canada CM Ltd., Vancouver, BC, Canada) and perlite (3:1 parts potting soil: perlite), and grown in a single growth chamber (10 h daylight, 26°C day:17°C night). The two mycorrhizal treatments were prepared by combining the soil mixture with live or autoclaved mycorrhizal inoculum consisting of a combination of root fragments and spores. We obtained inoculum of cosmopolitan fungal species known to associate with *Asclepias syriaca* (Vannette and Hunter, 2011) including *Rhizophagus intraradices, Funneliformis mosseae, G. aggregatum*, and *Claroideoglomus etunicatum* (Stockinger et al., 2009) from Mycorrhizal Applications (Grants Pass, OR, USA) and *Claroideoglomus etunicatum* cultured on the roots of *Sorghum* plants. Inocula from both sources were thoroughly mixed and made up 1/6 of the total pot volume, between layers of sterile soil. Although these species were not isolated from the rhizosphere of *Asclepias*, they have been found in forests, grassland, wetlands, and arable fields (Öpik et al., 2006), habitats in which all *Asclepias* species used here are also found. Seedlings were planted with either mycorrhizal fungi or autoclaved inoculum, watered *ad libitum*, and grown in a growth chamber for 12 weeks.

### Harvest and cardenolide analysis

Plants were harvested after 12 weeks of growth. Nearly all plants received damage by sciarid fly larvae present in the growth chamber (Vannette and Rasmann, 2012). To quantify the number of larvae in roots, we placed potato discs near plant roots for 3 days and counted fly larvae as they colonized the discs. Following larval counts, plant tissue was harvested, separated into AG and BG tissues, dried at 40°C and weighed. We verified mycorrhizal colonization by staining roots and examining them for mycorrhizal structures [data presented in Vannette and Rasmann (2012)].

To quantify the expression of cardenolides in AG and BG tissue, we used previously described methods to analyze cardenolide concentrations in foliar and root tissue (Zehnder and Hunter, 2007). Fine root and foliar tissues for each plant were ground separately, extracted in methanol for 1 h, and the subsequent extract dried and re-suspended in methanol with digitoxin as an internal standard. Each sample was filtered through a 0.2 μm filter and cardenolide compounds separated using UPLC (Waters Inc.) on an Acquity BEH C18 column (1.7 μm, 2.1 × 50 mm, Waters, Milford, MA, USA). Each 2 μl injection was eluted at a constant flow of 0.7 ml/min with a gradient of acetronitrile (ACN) and water, maintained at 20% ACN for 3 min, increasing to 45% ACN through the 9 min run. Peaks were detected by a diode array detector at 218 nm, and absorbance spectra were recorded from 200 to 400 nm. Peaks with symmetrical absorbance between 218 and 222 nm were quantified as cardenolides (Malcolm and Zalucki, 1996). Cardenolide concentrations were calculated using the digitoxin internal standard and initial sample mass, and total cardenolides were calculated as the sum of individual peaks.

### Calculations and statistical analyses

**Figure 1:**
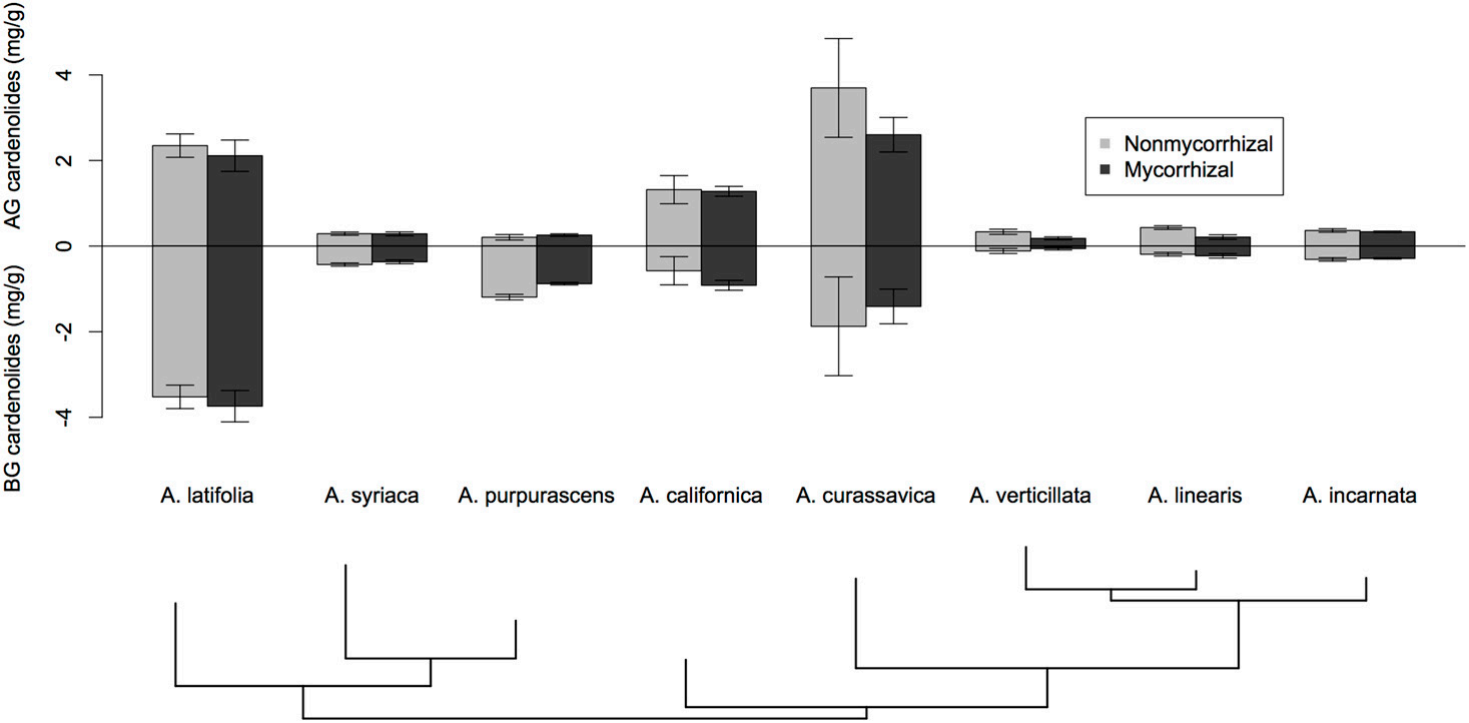
**The effect of AMF inoculation on cardenolide concentrations above- and below-ground among eight species of *Asclepias*.** Bars are mean ± 1SE. Bottom of figure shows the phylogenetic relatedness of *Asclepias* species used in this study. Phylogram pruned after Fishbein et al. (2011).

We assessed whether inoculation with mycorrhizal fungi influenced several indices of cardenolide expression. For both AG and BG tissues, we calculated cardenolide diversity and average polarity (Lefevre et al., 2010; Rasmann and Agrawal, 2011b). We used Shannon's index to calculate cardenolide diversity. We calculated cardenolide non-polarity following Sternberg et al. (2012); evidence suggests that non-polar cardenolides and a high diversity of cardenolides produce higher toxicity to a variety of organisms than do polar cardenolides or low diversity mixes (Fordyce and Malcolm, 2000; Zehnder and Hunter, 2007; Sternberg et al., 2012). Non-polarity was calculated for each sample by summing the relative peak areas multiplied by each peaks' retention time. Response variables were assessed for normality and homogeneity of variance and log-transformed if needed to reduce heteroscedasticity. Using Two-Way ANOVA, we examined if *Asclepias* species, AMF treatment, or their interaction affected each response variable, including total cardenolide concentration, diversity, and polarity. To control for the potential effect of root-feeding fly larvae on plant defense expression, we included larval density (the number of larvae divided by the total root biomass per plant) in the model as a covariate; it was never a significant variable, and was dropped from all subsequent analyses. Based on preliminary results (Figure 1), we explored whether the effects of AMF on cardenolide expression AG varied between phylogenetic clades. We used *post-hoc* contrasts comparing the effects of AMF on the five species in the *A. curassavica* clade to the effects of AMF on the clade containing *A. syriaca*. Contrasts were coded and tested using the multcomp package in R.

To assess if AMF influenced the composition (i.e., identity and relative abundance) of cardenolide compounds AG or BG, we used non-metric multidimensional scaling (NMDS) implemented in the vegan package (Oksanen et al., 2012) in R v 2.15.2 (Team, 2012). Differences in cardenolide composition among species, AMF treatment, and their interaction were tested using a permutational ANOVA (PERMANOVA), using the adonis function in the vegan package (Oksanen et al., 2012). The Bray–Curtis metric was used to calculate dissimilarity among samples for both the NMDS and PERMANOVA, although results were robust to other distance metrics. We also assessed the relationship between cardenolide expression in root and foliar tissue by regressing species' means for total AG and BG cardenolides using a Phylogenetic Least Squares (PGLS) framework. We estimated phylogenetic signal in average AG and BG growth and defense traits by calculating lambda values (Table S1), and phylogenetic regressions were estimated using the pgls function implemented in caper (Orme et al., 2012).

To explore if AMF influenced the distribution of cardenolides between plant roots and shoots, we calculated the root:shoot ratio of total cardenolide concentrations and used two-way ANOVA to examine the effects of *Asclepias* species, AMF treatment, and their interaction on root: shoot ratio of plant biomass and cardenolide concentrations. We also examined if AMF affected (a) plant biomass and (b) cardenolide concentration similarly AG and BG. For both mycorrhizal and non-mycorrhizal plants, we calculated mean values for AG and BG biomass and total cardenolide concentration for each species, then took the difference between mycorrhizal and non-mycorrhizal values. The difference in AG biomass was regressed against the difference in BG biomass (and similarly AG against BG cardenolides) using the PGLS framework. In this way, we could distinguish the effect of AMF inoculation on trait expression from variation arising from relatedness among species. Finally, to examine if AMF affected hypothesized growth-defense tradeoffs among plant individuals, we regressed plant biomass against whole-plant cardenolide concentration, and examined if AMF inoculation influenced the strength or direction of this relationship.

## Results

**Figure 2:**
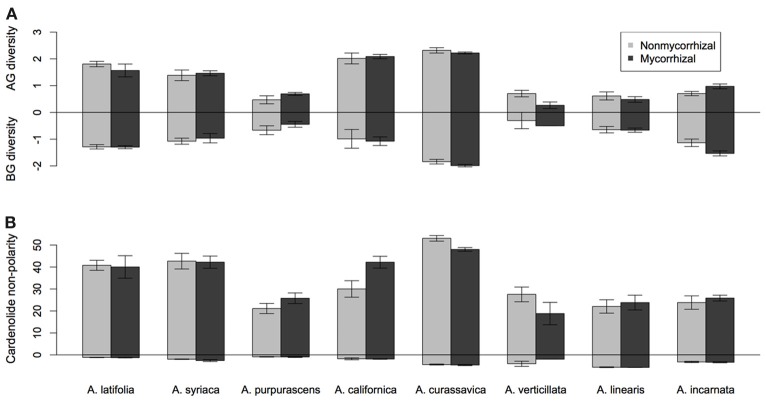
**Effect of AMF inoculation on above-ground (AG) and below-ground (BG) metrics of (A) cardenolide diversity (H), and (B) non-polarity, a measure of cardenolide toxicity (see text for details).** Bars display the mean ± 1SE for each species.

*Asclepias* species varied substantially in their expression of cardenolides in both foliar tissue [Figure 1; Species *F*_(7, 117)_ = 21.26, *p* < 0.001] and in fine roots [Figure 1; Species *F*_(7, 113)_ = 21.08, *p* < 0.0001]. Mycorrhizal inoculation affected the expression of cardenolides AG in a species-specific fashion [Figure 1; Species × AMF *F*_(7, 117)_ = 2.25, *p* = 0.034]. *Post-hoc* contrasts indicated that AMF inoculation decreased cardenolide concentration AG to a greater extent in the *A. curassavica* clade (5 species) than in the clade containing *A. syriaca* [3 species; *t* = 2.62, *p* = 0.009; Figure 1]. In contrast, AMF tended to increase root cardenolide concentration across all species to a small extent, but this effect did not vary significantly among *Asclepias* species [Figure 1; AMF treatment *F*_(1, 149)_ = 5.46, *p* = 0.02; Species × AMF *F*_(7, 149)_ = 1.05, *p* = 0.39]. *Asclepias* species varied in the diversity of cardenolides expressed, both AG and BG [Figure 2A; AG Species *F*_(7, 114)_ = 0.41, *p* < 0.001; BG Species *F*_(7, 128)_ = 17.21, *p* < 0.001]. Similarly, *Asclepias* species varied in cardenolide polarity both AG and BG [Figure 2B; AG Species *F*_(7, 117)_ = 10.44, *p* < 0.001; BG Species *F*_(7, 128)_ = 76.71, *p* < 0.001]. Notably, the cardenolides expressed by all *Asclepias* species are much more non-polar AG than they are BG (Figure 2B). Mycorrhizal inoculation did not affect cardenolide diversity either AG or BG [Figure 2A; AG AMF *F*_(1, 114)_ = 0.08, *p* = 0.77; BG AMF *F*_(1, 128)_ = 0.26, *p* = 0.61]. Mycorrhizal inoculation increased cardenolide non-polarity in AG plant tissue, although this change was driven largely by changes in *A. californica* and *A. purpurascens* [Figure 2B; AMF *F*_(1, 114)_ = 4.29, *p* = 0.04], but inoculation did not significantly affect the polarity of cardenolides in root tissue [Figure 2B; AMF *F*_(1, 121)_ = 0.90, *p* = 0.34].

**Figure 3:**
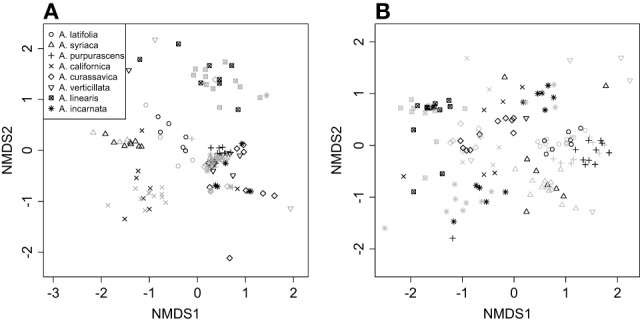
**NMDS plot illustrating variation in the composition of (A) above-ground foliar and (B) below-ground root cardenolides among eight milkweed species (*Asclepias spp.*), and the effects of AMF on cardenolide composition.** Points represent plant individuals, where black points are mycorrhizal plants and gray points are non-mycorrhizal points.

Cardenolide composition (the identity and relative abundance of molecular types) differed dramatically between roots and shoots, so were analyzed separately (Table S2). NMDS illustrated that *Asclepias* species varied in the composition of cardenolides expressed AG, and that AMF colonization shifted foliar cardenolide composition in a species-specific fashion [Figure 3A; PERMANOVA Species *F*_(7, 114)_ = 18.08, *P* < 0.001; AMF *F*_(1, 114)_ = 2.46, *P* = 0.021; Species × AMF *F*_(7, 114)_ = 1.65, *P* = 0.007]. Similarly, *Asclepias* species varied in cardenolide composition BG, and AMF also shifted cardenolide composition in a species-specific fashion [Figure 3B, PERMANOVA Species *F*_(7, 121)_ = 18.3, *P* < 0.001; AMF *F*_(1, 121)_ = 1.55, *P* = 0.08; Species × AMF *F*_(7, 121)_ = 1.24, *P* = 0.04].

Pagel's lambda indicated that most species traits exhibit some level of phylogenetic signal (Lambda close to 1), with the exception of AG biomass and BG cardenolide diversity (Table S1). However, 95% confidence intervals usually included both zero and one, likely due to the small number of species used to estimate lambda values.

**Figure 4:**
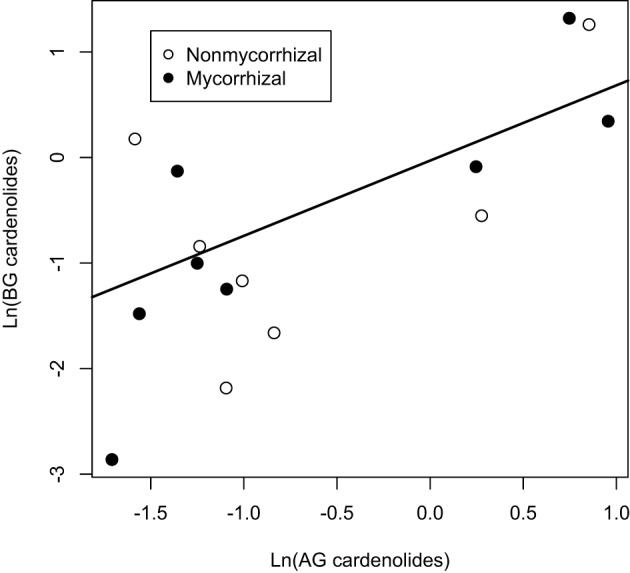
**Regressions between above (AG) and below-ground (BG) cardenolides among 8 species of *Asclepias*.** Points represent the mean level of cardenolides in plants that were inoculated with AMF or sterilized inoculum (non-mycorrhizal). Line represents PGLS best-fit line.

**Figure 5:**
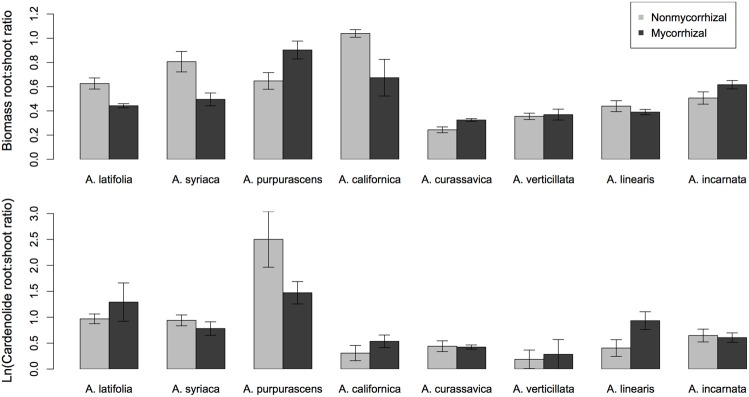
**Effects of mycorrhizal inoculation on (A) root:shoot biomass ratio, and (B) ratio of total cardenolide concentrations in roots:foliar tissue, among eight species of *Asclepias*.** Cardenolide ratios were log-transformed after calculation.

Among *Asclepias* species, the expression of cardenolides AG and BG was positively correlated (Figure 4), even when phylogeny was taken into account [PGLS regression *F*_(2, 6)_ = 7.8, *p* = 0.02]. This relationship did not differ between mycorrhizal and non-mycorrhizal plants (Figure 4). We also examined the effects of mycorrhizal inoculation on root:shoot biomass ratio. While root:shoot allocation varied among species [*F*_(7, 112)_ = 11.63, *p* < 0.001], the effect of AMF inoculation on root:shoot ratio varied in direction and magnitude among species [Figure 5A; Species × AMF *F*_(7, 112)_ = 5.94, *p* < 0.001]. *Asclepias* species varied in the ratio of root:foliar cardenolides [Species *F*_(7, 110)_ = 4.95, *p* < 0.001], and mycorrhizal inoculation also influenced the ratio of total cardenolides in roots to those in plant leaves in a species-specific fashion [Figure 5B, Species × AMF *F*_(7, 110)_ = 5.44, *p* = 0.008].

**Figure 6:**
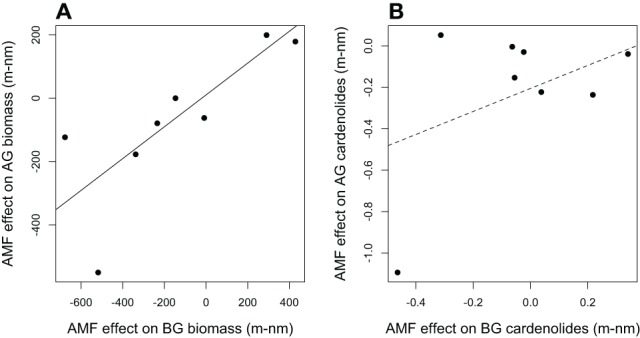
**The relationship between the effects of AMF on *Asclepias* (A) above-ground (AG) and below-ground (BG) biomass and (B) total cardenolide concentration.** Effects are calculated as mycorrhizal (m) minus non-mycorrhizal (nm) values. Each point represents the average effect of AMF for one of eight species of *Asclepias*. PGLS regression lines are shown, and the solid line indicates that the regression is significant at *p* < 0.05.

In addition, we used the PGLS framework to explore the relationship between AMF effects on biomass and cardenolides in foliar and root tissue (Table S1). The effect of AMF inoculation on shoot biomass was strongly positively correlated with the effect on root biomass among plant species, even when phylogenetic relatedness was accounted for [Figure 6A; PGLS *F*_(2, 6)_ = 12.8, *R*^2^ = 0.62, *p* = 0.01]. However, the effect of AMF on foliar cardenolide concentration was unrelated to its effect on roots [Figure 6B; *F*_(2, 6)_ = 1.5, *R*^2^ = 0.06, *p* = 0.26].

**Figure 7:**
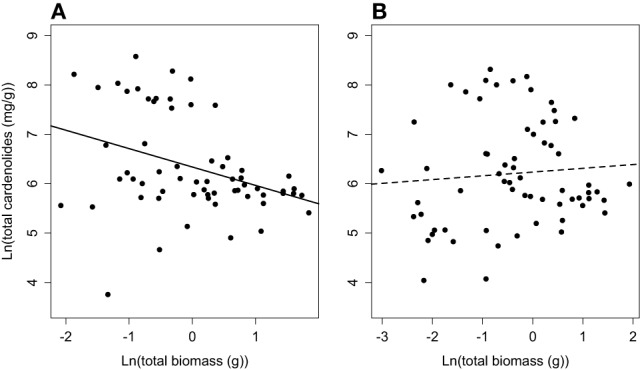
**Phenotypic correlation between total plant biomass and whole-plant cardenolide concentration for (A) non-mycorrhizal and (B) mycorrhizal *Asclepias plants***.

Finally, we identified a growth-defense tradeoff among non-mycorrhizal plants. Specifically, total plant biomass and whole-plant cardenolide concentration were negatively correlated (Figure 7A), explaining 11% of the variation in whole-plant cardenolide concentration [*F*_(1, 60)_ = 8.62, *R*^2^ = 0.11, *p* = 0.004]. However, when we assessed the same relationship in mycorrhizal plants, no significant relationship was detected [*F*_(1, 62)_ = 0.41, *R*^2^ = 0, *p* = 0.52] (Figure 7B). A similar trend was detected at the whole-plant level, but was not statistically significant (data not shown).

## Discussion

Our results demonstrate that mycorrhizal fungi differentially affect the expression and composition of AG and BG plant chemical defense traits, and that in large part, the effects of AMF inoculation are species-specific across the eight species of *Asclepias* examined here. In addition, we found evidence that AMF inoculation may alleviate the phenotypic tradeoff between plant growth and defense. Taken together, these results confirm that AMF can affect the traits that mediate interactions between plants and herbivores. However, variation in the magnitude and direction of responses to AMF within this single genus of plants was notable. Examining which effects of AMF are consistent among species and which vary may improve our ability to generalize about the multitrophic effects of AMF across species.

Surprisingly, the plants whose foliar cardenolide concentrations were affected most by AMF (e.g., *A linearis*, *A. verticillata*) showed little response in their root cardenolide concentrations to AMF colonization. Other species (e.g., *A. purpurascens* and *A. incarnata*) showed the reverse trend (Figures 1A,B). This suggests that not only do plant species vary in phenotypic response to inoculation with AMF (Klironomos, 2003), but plant species also vary in the tissues that are most responsive to inoculation with AMF. Variation among plant species in their responses to AMF can result from differences between coevolved and novel combinations of mutualists (Klironomos, 2003; Johnson et al., 2010) and shared evolutionary history among plants (Reinhart et al., 2012). Phylogenetic relatedness could explain some variation seen in our experiment: estimates of lambda were close to one for most cardenolide traits (Table S1), similar to previously documented patterns (Agrawal and Fishbein, 2008). Furthermore, the effect of AMF on AG cardenolide expression differed among clades within the *Asclepias* genus. However, the limited number of plant species included in this study hampered our ability to detect a significant phylogenetic signal in most plant traits. We suggest two non-mutually exclusive explanations for the variation among species in their phenotypic responses to AMF that we observed in our current study. First, *Asclepias* species may differentially associate with a subset of the AMF species available, as has been demonstrated with two forb species growing in grassland systems (Vandenkoornhuyse et al., 2002), and AMF species or communities may exert differential effects on plant phenotype (Bennett and Bever, 2007; Vannette and Hunter, 2011). Indeed, using the same plant species and inoculum, we found strong variation among *Asclepias* species in the number of arbuscules colonizing the roots, independent of phylogeny (Vannette and Rasmann, 2012), which may indicate differential association between *Asclepias* species and AMF. Second, plant species may vary in their phenotypic response to inoculation with the same fungal species because of differential resource transfer (Johnson et al., 2010), variation in hormonal or signaling response to colonization, or variation in plant ability to limit carbon flow to fungi (Grman, 2012). However, the variation in phenotypic response among *Asclepias* species indicates that, despite strong effects of mycorrhizal fungi on chemical defense in some species, predicting AMF-induced changes in plant resistance traits across taxa may prove difficult. An examination of the nutrient benefits transferred among partners, or other plant physiological traits may prove helpful in forming predictions for future work.

Given the demonstrated importance of AMF to the induced defense responses of plants (Kempel et al., 2010; Barber, 2012), it is likely that AMF-mediated changes in induction may also be important in resistance to herbivory among *Asclepias* species. Future work should examine if mycorrhizal fungi affect induction in a similar way among *Asclepias* species, and if AMF-plant relationships interact with latitudinal patterns in the evolution of induction (Rasmann and Agrawal, 2011b).

In addition to species-level variation in response to AMF, we observed differential effects of AMF on the expression of chemical compounds BG and AG. The effects of AMF on AG cardenolide concentration and composition varied among *Asclepias* species, but AMF inoculation had statistically similar and weak effects among species on BG cardenolide concentration and composition. However, AMF effects on cardenolide composition were species-specific both AG and BG (Figure 3). In addition, although the effects of AMF on AG and BG biomass were strongly positively correlated, AMF effects on the total concentration of cardenolides AG and BG were unrelated (Figure 6). This suggests that different mechanisms control plant biomass and secondary metabolite allocation when associating with AMF in *Asclepias*. Additionally, cardenolide profiles differed dramatically between foliar and root tissues, confirming previous results in milkweed (Nelson et al., 1981; Rasmann and Agrawal, 2011b; Agrawal et al., 2012) and broad patterns of defense across plant species overall (van Dam, 2009). Although cardenolides can be synthesized in shoot tissue (Groeneveld et al., 1990), the major site of synthesis is not well-understood, and it is possible that cardenolide biosynthesis may be ultimately fine-tuned in different plant organs (Manson et al., 2012). Despite the notable species-specific variation that we recorded, the differential effects of AMF on AG and BG resistance expression suggest a mechanism by which root and shoot feeders respond differently to mycorrhizal inoculation, as has been shown previously (Gange, 2001). Although we have already demonstrated that AMF convey protection against BG feeders (Vannette and Rasmann, 2012), future work should examine differences in effects of AMF between AG and BG herbivores. Investigating the role of AMF on insects that display differential mother-offspring feeding strategies, as seen in milkweed specialist *Tetraopes* beetles, may be an informative avenue to explore differential effects on AG and BG root feeders.

Cardenolides expressed in fine roots were considerably less non-polar than were cardenolides expressed in foliage (Figure 2B), despite the similar concentration and diversity of cardenolides in AG and BG tissues (Figures 1A,B, 2A). Non-polar cardenolides are generally considered more toxic than are polar cardenolides, because non-polar forms may pass more easily across cell membranes (Fordyce and Malcolm, 2000). So our results suggest that cardenolides in fine roots occur at equal concentrations, but in less toxic forms, than those in leaves. This finding contrasts with results described by Rasmann and Agrawal (2011b), who found comparable cardenolide polarity in AG and BG tissues. However, only fine roots (the site of AMF colonization) were used in the current analysis, whereas the previous study also included rhizome, which is stem tissue. Work from multiple systems has demonstrated that main and storage roots are often well-protected compared to fine roots (van Dam, 2009), which may suggest differential pressure by herbivores or differences in root development, longevity, or value to plants. In addition, this pattern may also indicate plant adaptation to association with fungal mutualists, which occurs in fine roots, but not rhizome. Microbial mutualists in fine roots may be susceptible to non-polar compounds, compete for carbon otherwise used for defense synthesis, or even receive such compounds for their own protection in the presence of fungivores (Duhamel et al., 2013).

Finally, mycorrhizal inoculation altered the putative growth-defense tradeoff that we documented in non-mycorrhizal plants. In previous work, we hypothesized that mycorrhizal fungi may modify growth-defense tradeoffs by either increasing nutrient acquisition by plants or increasing C cost at high levels of AMF abundance (Vannette and Hunter, 2011). Although we did not measure plant nutrient status in this experiment, our results lend tentative support to this hypothesis. Indeed, the general difficulty in identifying growth-defense tradeoffs in natural populations may be due to plant associations with herbivores or mutualists, including mycorrhizal fungi that modify predicted growth-defense relationships (Herms and Mattson, 1992).

## Conclusions

The results described here demonstrate that despite pervasive effects of AMF on plant resistance expression, significant variation exists among congeners in their responses to mycorrhizal colonization. As a result, we suggest that exploring the ecological or molecular correlates of such variation may lead to a better understanding of how BG microorganisms influence multitrophic interactions.

## Conflict of interest statement

The authors declare that the research was conducted in the absence of any commercial or financial relationships that could be construed as a potential conflict of interest.
