# Supplementary material for: Arbuscular mycorrhizal fungi alter above- and below-ground chemical defense expression differentially among Asclepias species
Source: Front Plant Sci. 2013 Sep 19;4:361. doi: 10.3389/fpls.2013.00361 (PMC3776932; doi:10.3389/fpls.2013.00361)

**Supplementary Material**

**S1**. Pagel’s lambda values calculated for traits expressed by eight species of *Asclepias*. Values close to one indicate phylogenetic conservation of traits, where more closely related species exhibit similar trait values. Mean trait values were used, and lambda values were estimated using the pgls function in the R package caper. Lambda indicates Pagel’s lambda estimate, and P-values indicate significance tests for comparisons against lower and upper bounds on lambda.

| **Trait (species averages)** | **Lambda** | **L.bound** | **P-value** | **U.bound** | **P-value** | **95% CI** |
| --- | --- | --- | --- | --- | --- | --- |
| AG biomass | 0 | 0 | 1 | 1 | 0.007 | NA,0.74* |
| ln(Total AG cardenolides) | 1 | 0 | 0.15 | 1 | 1 | NA |
| ln(Total BG cardenolides) | 1 | 0 | 0.1 | 1 | 1 | NA |
| AG cardenolide diversity | 1 | 0 | 0.13 | 1 | 1 | NA |
| AG cardenolide polarity | 1 | 0 | 0.39 | 1 | 1 | NA |
| BG cardenolide diversity | 0 | 0 | 1 | 1 | 0.1 | NA |
| BG cardenolide polarity | 0.65 | 0 | 0.15 | 1 | 0.19 | NA |
| Root:Shoot biomass | 1 | 0 | 0.12 | 1 | 1 | NA |
| Root:Shoot cardenolides | 1 | 0 | 0.43 | 1 | 1 | NA |
| **Regressions** |  |  |  |  |  |  |
| AG Cardenolides~  BG Cardenolides | 1 | 0 | 0.1 | 1 | 1 | NA |
| Difference in AG biomass~  Difference in BG biomass | 0 | 0 | 1 | 1 | 0.08 | NA |
| Difference in AG cardenolides ~ Difference in BG cardenolides | 1 | 0 | 0.5 | 1 | 1 | NA |

- upper bound, but not lower bound was estimated with confidence.

S2. Mean cardenolide concentration (mg/g) for each cardenolide peak (isolated by retention time, min) in the a) foliage and b) fine roots of eight *Asclepias* species. Concentrations are the means of between 14 and 22 samples, depending upon species (see text for details). Labels indicate the relative time of each peak, relative to the internal standard (digitoxin), where the peak’s retention time is divided by that of digitoxin.

a.


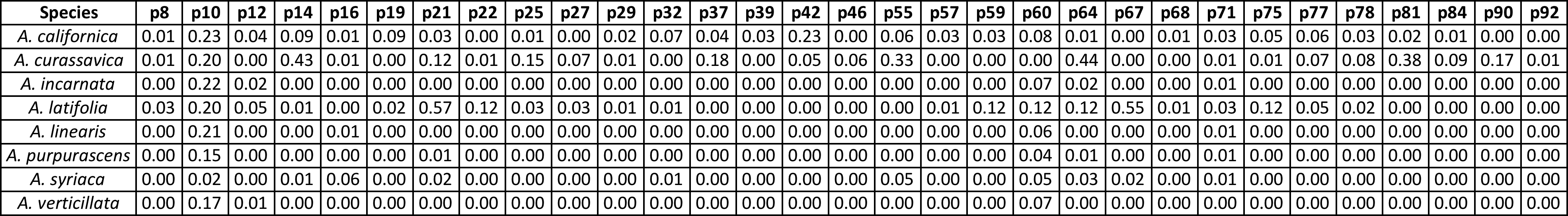


b.


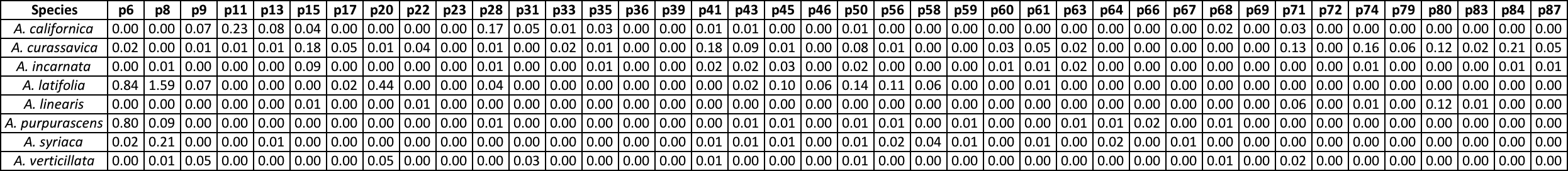

Supplement: Supplementary file 1 [file DataSheet1.DOCX]
